# CEA and CA-19-9 Dynamics Associate with Survival in Regorafenib-Treated Metastatic Colorectal Cancer: A Real-World Analysis

**DOI:** 10.3390/jcm15072599

**Published:** 2026-03-29

**Authors:** Merih Yalçıner, Mehmet Berk Örüncü, Mehmet Kayaalp, Efe Cem Erdat, Engin Eren Kavak, Güngör Utkan

**Affiliations:** Department of Medical Oncology, Ankara University Faculty of Medicine, 06100 Ankara, Türkiye; mehmetberkoruncu@gmail.com (M.B.Ö.); kayaalpmehmet2728@gmail.com (M.K.); cemerdat@gmail.com (E.C.E.);

**Keywords:** metastatic colorectal cancer, regorafenib, CEA, CA-19-9, biomarkers, real-world evidence, survival analysis

## Abstract

**Background:** Regorafenib is a standard late-line treatment for refractory metastatic colorectal cancer (mCRC), yet clinical outcomes remain heterogeneous. Identifying accessible biomarkers to predict therapeutic benefit is crucial for balancing efficacy and toxicity. This study evaluated the prognostic value of early dynamic changes in carcinoembryonic antigen (CEA) and carbohydrate antigen 19-9 (CA-19-9). **Methods:** We conducted a retrospective real-world analysis of 61 heavily pretreated mCRC patients receiving regorafenib. Tumor markers were assessed at baseline and after the third cycle (approximately 12 weeks). Systematic threshold optimization using Cox proportional hazards regression and Akaike Information Criterion (AIC) was performed to identify optimal percentage change cutpoints for predicting survival. **Results:** The optimal thresholds for defining unfavorable marker increases were +14.6% for CEA and +23.5% for CA-19-9. Patients with CEA changes below the cutoff demonstrated significantly superior progression-free survival (PFS) (median 6.1 vs. 4.0 months, *p* = 0.012) and overall survival (OS) (11.05 vs. 6.0 months, *p* = 0.035). CA-19-9 changes below the cutoff were associated with improved PFS (*p* = 0.022) but did not reach statistical significance for OS (*p* = 0.23). In multivariable analysis, neither marker retained independent significance, likely due to collinearity. **Conclusions:** Early dynamics of routine tumor markers are prognostic in regorafenib-treated mCRC. specifically, a CEA increase of <14.6% significantly predicts improved survival outcomes. These findings support the utility of serial marker monitoring in real-world practice, though prospective validation is warranted.

## 1. Introduction

Colorectal cancer (CRC) ranks as the third most commonly diagnosed malignancy worldwide and the second leading cause of cancer-related mortality in 2020, representing a substantial global health burden [1]. Despite significant advances in diagnostic techniques and novel targeted therapeutic options and high survival rates in early-stage disease, metastatic progression occurs in approximately one-third of CRC patients during follow-up, resulting in a marked decline in 5-year overall survival (OS) [2,3,4]. Current treatment paradigm for metastatic colorectal cancer (mCRC) includes fluoropyrimidine-based chemotherapy, with the addition of targeted therapies including anti-vascular endothelial growth factor (VEGF) and anti-epidermal growth factor receptor (EGFR) agents [5], BRAF inhibitors [6], and immune checkpoint inhibitors for microsatellite instability-high (MSI-H) tumors [7], with treatment selection increasingly guided by molecular profiling. However, the majority of patients eventually develop resistance to standard therapies, necessitating late-line treatment options.

Regorafenib, an oral multi-kinase inhibitor targeting VEGFR and other kinases involved in angiogenesis and tumor proliferation, has established efficacy in refractory mCRC, with the CORRECT and CONCUR trials demonstrating OS improvements, leading to its role as a standard late-line treatment option [8,9]. However, as with other multikinase inhibitors, regorafenib may cause treatment-emergent adverse events that can impair quality of life, including hand-foot skin reaction, fatigue, hypertension, and diarrhea. The large-scale, single-arm CONSIGN study reported a median progression-free survival (PFS) of 2.7 months with a manageable toxicity profile, though dose reductions were required in 46% of patients due to treatment-emergent adverse events [10]. Real-world evidence from the CORRELATE study further validated these findings in Asian populations, demonstrating a median PFS of 2.2 months, with 72% of patients initiating treatment at reduced doses and 50% requiring dose interruptions, highlighting the practical challenges of balancing efficacy with tolerability in routine clinical practice [11]. Despite the survival benefits, clinical outcomes remain heterogeneous, with considerable variability in treatment response and toxicity profiles. Therefore, several studies have investigated potential clinical parameters and biomarkers to identify patients most likely to benefit from regorafenib treatment. These studies included clinical parameters such as tumor burden, metastatic sites, and tumor growth rate [12], prognostic scoring systems incorporating number or metastasis sites, liver function tests and inflammatory markers [13,14], and biochemical markers including early changes in CA-19-9 [15]. Additionally, emerging biomarkers such as circulating methylated DNA have shown promise in predicting treatment response [16].

Tumor markers CEA and CA-19-9 are among the most widely utilized biomarkers in CRC management, routinely measured during treatment monitoring due to their accessibility, low cost, and ability to be assessed more frequently than radiographic imaging [17,18,19,20]. However, the predictive value of dynamic on-treatment changes specifically during regorafenib therapy is not clearly defined. In this retrospective real-world study, we systematically evaluated CEA and CA-19-9 dynamics at two predefined time points (baseline, after third cycle) during regorafenib treatment to identify clinically meaningful early marker responses, as well as relevant clinical and pathological findings that predict survival benefit in heavily pretreated mCRC patients.

## 2. Materials and Methods

### 2.1. Study Design and Patient Population

This retrospective cohort study included patients with metastatic colorectal cancer (mCRC) treated with regorafenib at our tertiary cancer center between 10 June 2015–27 June 2025. Eligible patients had histologically confirmed colorectal adenocarcinoma with measurable metastatic disease, disease progression after ≥2 prior systemic chemotherapy regimens, ECOG performance status 0–2, and adequate hepatic and renal function. All patients had microsatellite-stable (MSS) tumors and prior exposure to fluoropyrimidine-based chemotherapy with or without oxaliplatin and irinotecan, as well as prior anti-VEGF therapy. Inclusion required baseline carcinoembryonic antigen (CEA) and carbohydrate antigen 19-9 (CA-19-9) measurements. The institutional ethics committee approved this study, which was conducted in accordance with the Declaration of Helsinki.

### 2.2. Data Collection and Tumor Marker Assessment

Comprehensive clinical data were extracted from electronic medical records, including age, sex, primary tumor location (right-sided: cecum to transverse colon; left-sided: splenic flexure to rectum), RAS/BRAF mutational status, metastatic sites, prior treatment history, baseline laboratory parameters, and ECOG performance status. Serum CEA (normal reference < 5 ng/mL) and CA-19-9 (normal reference < 37 U/mL) were measured using standardized immunoassay platforms at predefined timepoints: baseline (within 2 weeks prior to treatment initiation) and after the third regorafenib cycle (approximately 12 weeks post-initiation, IQR: 10–14 weeks. Radiological response evaluations (computed tomography or magnetic resonance imaging) were performed according to institutional standard of care, typically every 8 to 12 weeks, or sooner if clinically indicated by symptomatic deterioration. Disease progression was determined by the treating physicians based on the real-world application of RECIST v1.1 criteria in conjunction with clinical assessment.

### 2.3. Tumor Marker Threshold Optimization and Response Definitions

Percentage changes in CEA and CA-19-9 from baseline to third cycle were calculated. To identify optimal clinically meaningful thresholds, systematic threshold optimization was performed using proportional Cox hazards regression. For each marker, percentage change cutpoints ranging from −50% to +50% (in 5% increments) were evaluated. For each threshold, proportional Cox regression models were fitted to calculate hazard ratios (HR) and 95% confidence intervals (CI) for progression-free survival (PFS) and overall survival (OS). Model fit and predictive performance were assessed using Akaike Information Criterion (AIC), with lower AIC values indicating superior model performance.

Optimal cutpoints were selected based on: (1) minimum AIC values for PFS and OS models, (2) statistical significance (*p* < 0.05) of hazard ratios, (3) magnitude of HR separation between favorable and unfavorable groups, (4) clinical interpretability, and (5) adequate sample size (≥10 patients) in each stratum. Proportional hazards assumptions were verified using scaled Schoenfeld residuals. Threshold optimization identified CEA 14.6% increase and CA-19-9 23.5% increase as optimal cutpoints. Patients were stratified as favorable (below cutoff) or unfavorable (above cutoff) based on these thresholds.

Cutoff stability was evaluated using two approaches. First, bootstrap resampling (1000 iterations with replacement) was performed, with the full threshold optimization procedure repeated in each resample. The proportion of bootstrap samples selecting a cutoff within ±5% of the original value was calculated, along with the median, mean, interquartile range, and 95% bootstrap confidence interval of the selected cutoffs. Second, leave-one-out cross-validation (LOOCV) was performed, in which the optimal cutoff was re-derived from the training set in each iteration and the held-out observation classified accordingly. Concordance between LOOCV-predicted and original classifications was reported.

### 2.4. Statistical Analysis

All analyses were performed using R version 4.4.1 (R Foundation for Statistical Computing, Vienna, Austria). Continuous variables are presented as median (interquartile range, IQR) for skewed distributions or mean ± standard deviation (SD) for normally distributed data; categorical variables are summarized as frequencies and percentages. Progression-free survival (PFS) was defined as time from regorafenib initiation to disease progression per RECIST v1.1 criteria, clinical deterioration, or death from any cause, whichever occurred first. Overall survival (OS) was defined as time from treatment initiation to death from any cause. Patients without documented progression (for PFS) or alive (for OS) at last follow-up were censored. Kaplan-Meier survival curves were generated and compared using log-rank tests; median survival times with 95% confidence intervals (CI) were calculated with risk tables at multiple timepoints. Cox proportional hazards regression was used to evaluate the continuous association between percentage change in each marker and survival outcomes, with hazard ratios reported per 10% increase. Univariable Cox proportional hazards regression assessed associations between clinical variables, tumor marker changes, and survival outcomes. Variables meeting *p* < 0.10 in univariable analysis and CEA and CA-19-9 cutoff analyses were entered into multivariable Cox models using backward elimination (threshold *p* < 0.05), with hazard ratios and 95% CI reported. Time-dependent ROC analysis at 6 months was performed to assess discriminatory accuracy of tumor markers. All statistical tests were two-sided with α = 0.05.

## 3. Results

A total of 61 patients with metastatic colorectal cancer received regorafenib during the study period (Table 1). The median age was 62 years (range 33–87), and 60.7% were male. The majority had left-sided primary tumors (63.9%), RAS mutations (65.5%), and liver metastases (72.1%). BRAF mutation was present in one patient. All patients had microsatellite-stable (MSS) tumors. This heavily pretreated cohort received regorafenib as third-line therapy in 59.0% and fourth-line or later in 41.0% of patients. All patients had received prior anti-VEGF therapy, and among RAS wild-type patients, 24.6% had received prior anti-EGFR therapy. Most patients (54.1%) received a starting dose of 80 mg with planned escalation, while 32.8% started at the standard 160 mg dose. Treatment-related adverse events occurred in 60.7% of patients, requiring dose reduction in 48.3% (Table 1).

The median observed follow-up time for the entire cohort was 8 months, which directly mirrors the overall survival times. The single patient still alive at the time of analysis had an ongoing follow-up time of 50 months.

Complete tumor marker data across both timepoints were available for all 61 patients (100%). Median baseline CEA was 56.5 ng/mL (IQR: 17.9–247), which increased after the third cycle to 99.8 ng/mL (IQR: 24.8–335; median change +76.6%). Median baseline CA-19-9 was 58.1 U/mL (IQR: 24.4–272), which increased after the third cycle to 113 U/mL (IQR: 33.7–475; median change +94.5%).

When evaluated as continuous predictors using Cox proportional hazards regression, each 10% increase in CEA percentage change was associated with a trend toward worse PFS (HR 1.004, 95% CI 1.000–1.008, *p* = 0.054) and a significant association with worse OS (HR 1.007, 95% CI 1.002–1.011, *p* = 0.002). For CA-19-9, each 10% increase was significantly associated with both worse PFS (HR 1.002, 95% CI 1.000–1.003, *p* = 0.015) and worse OS (HR 1.002, 95% CI 1.000–1.003, *p* = 0.019).

**Figure 1:**
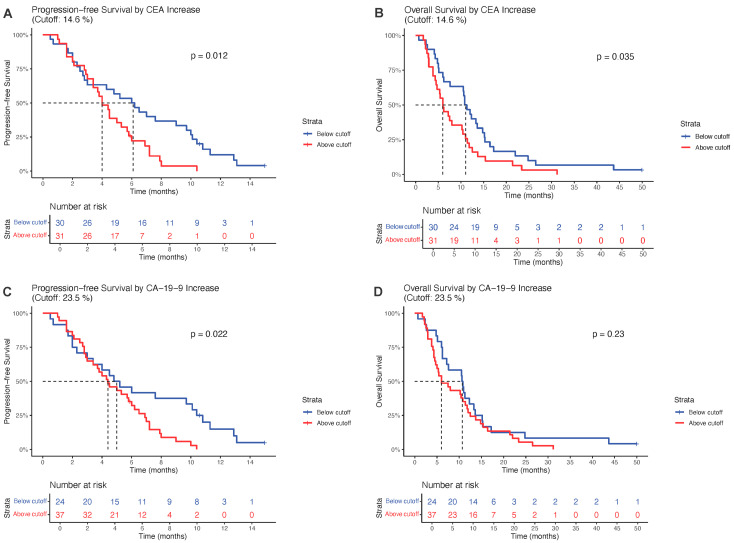
Progression-free survival (PFS) and overall survival (OS) stratified by optimized tumor marker cutoffs. (**A**) PFS by CEA threshold (14.6% increase; (**B**) OS by CEA threshold; (**C**) PFS by CA-19-9 threshold (23.5% increase; (**D**) OS by CA-19-9 threshold. Risk tables are displayed below each curve.

**Figure 2:**
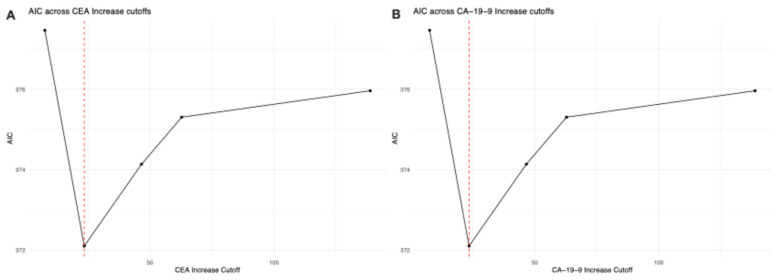
Threshold Optimization Analysis for CEA and CA-19-9. (**A**) CEA threshold optimization for PFS and OS; (**B**) CA-19-9 threshold optimization.

**Figure 3:**
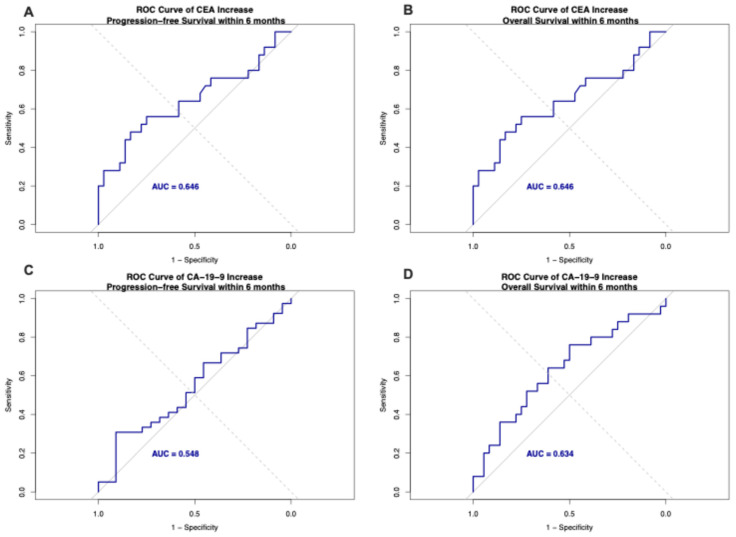
Time-Dependent Receiver Operating Characteristic (ROC) Curves at 6 Months. (**A**,**C**) progression-free survival and (**B**,**D**) overall survival at 6 months.

Proportional hazards Cox regression analysis identified optimal cutoff values of 14.6% increase for CEA and 23.5% increase for CA-19-9 (Figure 1). For CA-19-9, patients with increases below the cutoff demonstrated superior progression-free survival (PFS) with median 5.0 months (95% CI: 3.4–10.4) compared to those above cutoff at 4.4 months (95% CI: 3.3–6.2; *p* = 0.022). Overall survival (OS) trended favorably below cutoff at 10.7 months (95% CI: 7.2–13.6) versus 6.0 months (95% CI: 4.7–11.8) above cutoff, though this did not reach statistical significance (*p* = 0.23). For CEA, patients below the cutoff showed superior PFS at 6.1 months (95% CI: 3.0–10.0) compared to 4.0 months (95% CI: 3.4–5.8) above cutoff (*p* = 0.012). OS also favored the below-cutoff group at 11.05 months (95% CI: 7.6–15.2) versus 6.0 months (95% CI: 4.5–11.0) above cutoff (*p* = 0.035) (Figure 1). Akaike Information Criterion (AIC) values for model comparisons are presented in Figure 2. Time-dependent receiver operating characteristic (ROC) curves at 6 months for CEA and CA-19-9 predicting progression and overall survival are shown in Figure 3. At 6 months, CEA demonstrated comparable AUC values for OS (0.646) and PFS (0.646), while CA-19-9 showed AUC of 0.634 for OS and 0.548 for PFS.

**Figure 4:**
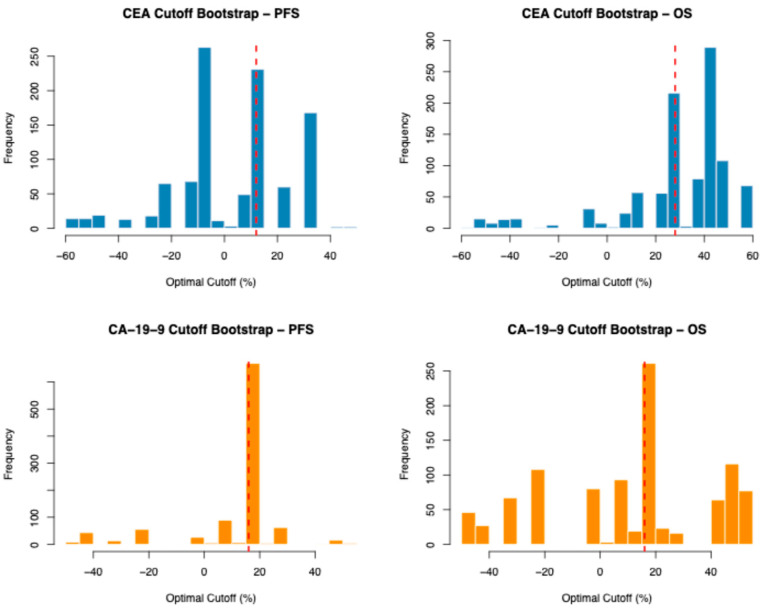
Bootstrap histograms of CEA and CA-19-9 levels for both progression-free and overall survival. Dashed red lines shows the optimal cutoff values.

Internal validation revealed variable cutoff stability depending on the marker and outcome. Bootstrap resampling (1000 iterations) demonstrated that the CA-19-9 threshold for PFS was the most stable, with 67.5% of resamples selecting a cutoff within ±5% of the original threshold and a narrow interquartile range. CEA cutoffs showed wider bootstrap distributions for both PFS and OS, with broad 95% confidence intervals spanning much of the tested range (Figure 4). These wide distributions are consistent with the known instability of data-driven threshold optimization in small samples and reinforce the importance of the continuous analysis as the primary finding. Leave-one-out cross-validation demonstrated high concordance between cross-validated and original patient classifications (CEA-PFS: 90.2%, CEA-OS: 91.8%, CA-19-9-PFS: 100%, CA-19-9-OS: 96.7%), indicating that individual patient classification was robust despite variation in the selected cutoff across iterations.

Univariable Cox regression analysis (Table 2) was performed to evaluate the prognostic significance of baseline clinical factors and tumor marker dynamics for both progression-free and overall survival.

For PFS, CEA above the optimized cutoff (14.6% increase) was significantly associated with worse outcomes (HR 2.06, 95% CI: 1.17–3.62, *p* = 0.013), as was CA-19-9 above cutoff (23.5% increase; HR 1.88, 95% CI: 1.05–3.36, *p* = 0.033). Gender, RAS/RAF mutational status, primary tumor sidedness, and regorafenib treatment line were not significantly associated with PFS in univariable analysis (all *p* > 0.05). In multivariable analysis including both tumor markers, neither CEA above cutoff (HR 1.71, 95% CI: 0.80–3.62, *p* = 0.164) nor CA-19-9 above cutoff (HR 1.32, 95% CI: 0.61–2.87, *p* = 0.478) retained statistical significance, suggesting potential correlation between these markers.

For OS, CEA above cutoff demonstrated borderline significance in univariable analysis (HR 1.67, 95% CI: 1.01–2.80, *p* = 0.050), whereas CA-19-9 above cutoff was not significantly associated with OS (HR 1.26, 95% CI: 0.75–2.12, *p* = 0.390). Gender, RAS/RAF mutational status, primary tumor sidedness, and treatment line showed no significant association with OS. In multivariable analysis, CEA above cutoff approached but did not reach statistical significance (HR 1.81, 95% CI: 0.93–3.54, *p* = 0.079), while CA-19-9 showed no association (HR 0.87, 95% CI: 0.44–1.71, *p* = 0.692).

Notably, conventional clinical prognostic factors including gender, RAS/RAF mutational status, primary tumor location, and treatment line were not independently predictive of either PFS or OS in this heavily pretreated cohort, suggesting that tumor marker dynamics may capture prognostic information not reflected by baseline clinical characteristics.

In univariable Cox regression analysis for progression-free survival (PFS), CEA above cutoff was associated with increased risk (HR 2.06, 95% CI: 1.17–3.62, *p* = 0.013) and CA-19-9 above cutoff showed similar association (HR 1.88, 95% CI: 1.05–3.36, *p* = 0.033). Gender, RAS/RAF mutational status, primary tumor sidedness, and treatment line were not significantly associated with PFS (all *p* > 0.05). In multivariable PFS analysis, CEA above cutoff remained marginally associated with worse PFS (HR 1.71, 95% CI: 0.80–3.62, *p* = 0.164), while CA-19-9 lost statistical significance (HR 1.32, 95% CI: 0.61–2.87, *p* = 0.478), (Table 2).

For overall survival (OS), univariable analysis demonstrated that CEA above cutoff approached significance (HR 1.67, 95% CI: 1.01–2.80, *p* = 0.050), whereas CA-19-9 above cutoff was not significantly associated with OS (HR 1.26, 95% CI: 0.75–2.12, *p* = 0.390). In multivariable OS analysis, CEA above cutoff remained associated with worse survival (HR 1.81, 95% CI: 0.93–3.54, *p* = 0.079), while CA-19-9 showed no association (HR 0.87, 95% CI: 0.44–1.71, *p* = 0.692). Gender, RAS/RAF status, primary tumor location, and treatment line were not independently predictive of OS in either univariable or multivariable analyses (Table 2).

## 4. Discussion

This real-world study systematically evaluated early tumor marker dynamics in heavily pretreated mCRC patients receiving regorafenib. As the primary analysis, continuous Cox regression demonstrated that on-treatment percentage changes in both CEA and CA-19-9 are linearly associated with survival outcomes, with CEA showing a particularly strong association with OS (HR 1.007 per 10% increase, *p* = 0.002). Restricted cubic spline analysis confirmed that these relationships are consistent with linearity (non-linearity *p* > 0.13 for all), supporting the biological interpretation that greater marker increases reflect a continuous gradient of unfavorable prognosis rather than a discrete threshold effect. As a secondary, clinically oriented analysis, threshold optimization using the maximally selected log-rank statistic identified clinically meaningful cutpoints for CEA (14.6% increase) and CA-19-9 (23.5% increase) from baseline to third cycle. Both tumor markers demonstrated prognostic significance in univariable analysis: CEA above threshold was significantly associated with inferior PFS (*p* = 0.013) and borderline significance for OS (*p* = 0.050), while CA-19-9 above threshold was significant for PFS (*p* = 0.033) but not OS (*p* = 0.390). However, neither marker retained independent prognostic significance in multivariable analysis, likely reflecting limited statistical power in this 61-patient cohort, as formal correlation testing revealed negligible association between CEA and CA-19-9 on-treatment dynamics (Spearman rho = 0.056, *p* = 0.67). Although the two markers are uncorrelated at the biomarker level, they may capture partially overlapping prognostic information at the survival outcome level through distinct biological mechanisms, attenuating individual effect estimates in a combined model with limited events. Notably, conventional clinical factors including gender, RAS/RAF mutational status, primary tumor location, and treatment line were not significantly associated with survival outcomes in this cohort. To our knowledge, this is the first study to combine continuous biomarker analysis with systematic threshold optimization specifically for regorafenib-treated mCRC patients.

The prognostic value of tumor marker dynamics in CRC has been demonstrated in other therapeutic contexts. Prior studies have shown that CEA kinetics combined with CA-19-9 provide prognostic information during chemotherapy [20], and perioperative longitudinal measurements of CEA, CA-19-9, and CA125 improve prognostic accuracy following curative resection [19]. However, evidence specifically evaluating tumor marker kinetics during regorafenib therapy remains limited.

Previous studies have identified various clinical and laboratory parameters associated with regorafenib outcomes, including tumor burden metrics, inflammatory markers, and baseline biochemical parameters. Some studies suggested that third and fourth line regorafenib treatment in mCRC is associated with longer response duration, which aligns with our study [21]. The analysis of the REGOTAS study identified AST > 40 IU/dL, CRP ≥ 1.0 mg/dL, ≥3 metastatic organ sites, and first-line chemotherapy duration < 18 months as the worst prognostic factors [13]. Similarly, another study emphasized low CEA, slow tumor growth and few metastatic sites as criteria for optimal patient selection [12] and another study comparing regorafenib vs. immunotherapy showed decreasing CEA levels associating with better survival outcomes [22]. In another study that included 555 mCRC patients treated with regorafenib in real-world clinical practice, multivariable analysis identified that higher body mass index, longer interval from metastatic diagnosis, and ECOG PS 0 predicted longer OS [23]. While these studies predominantly focused on baseline parameters, our approach evaluated marker dynamics across three distinct timepoints, providing insight into how tumor marker trajectories reflect evolving treatment response and disease biology during regorafenib therapy. A study of 146 patients identified early CA-19-9 decrease as an independent predictive factor [15], consistent with our findings. Other investigators have developed composite scoring systems such as the Cancer-Inflammation Prognostic Index (CIPI), which integrates inflammatory markers with clinical parameters to predict survival [14]. Our findings support the importance of inflammatory parameters in regorafenib outcomes.

Regarding RAS mutation status, our finding of independent adverse prognostic significance does not differ from some prior reports. Two previous studies reported that KRAS/NRAS mutations did not significantly impact regorafenib outcomes [23,24]. The exploratory analysis of the CORRECT trial examined circulating DNA and protein biomarkers in mCRC patients receiving regorafenib. Regorafenib demonstrated consistent clinical benefit across all patient subgroups regardless of KRAS mutational status, PIK3CA status, or circulating DNA concentration. Among plasma proteins assessed in 611 patients, none significantly predicted regorafenib efficacy [25]. These findings may reflect differences in sample size, patient populations, or treatment settings. Biologically, RAS mutations may confer resistance mechanisms to anti-angiogenic therapy through alternative signaling pathways, though this hypothesis requires further mechanistic investigation.

An important observation in our threshold-based analysis is the discrepancy between the early Kaplan-Meier curve separation for OS and the delayed separation for PFS when stratified by CEA threshold. Several factors may contribute to this pattern. First, PFS determination depends on radiographic documentation per RECIST v1.1, and variability in imaging intervals in this retrospective cohort may have introduced lag in progression detection, particularly during early treatment. OS, as an objective endpoint not subject to assessment timing, may therefore demonstrate a more immediate and consistent separation. Second, the earlier OS separation may reflect shorter post-progression survival in the unfavorable marker group, potentially driven by more aggressive disease biology or fewer effective subsequent treatment options in this heavily pretreated population. The continuous analysis supports this interpretation, as CEA showed a notably stronger association with OS (*p* = 0.002) than with PFS (*p* = 0.054), suggesting that CEA dynamics may better capture the overall disease trajectory than the timing of first documented progression. These observations should be interpreted cautiously given the small sample size and the inherent instability of Kaplan-Meier curves with limited numbers of patients at risk.

Beyond clinical parameters and routine tumor markers, emerging biomarkers have been explored to predict regorafenib efficacy. Circulating methylated DNA (cmDNA) has shown promise as both a prognostic and predictive marker, with high baseline and on-treatment cmDNA levels associated with shorter PFS and increased progression risk [16]. Similarly, analysis of soluble protein biomarkers identified higher baseline levels of OPN, VCAM-1, and PDGF-AA as potential predictors of regorafenib benefit [26]. While these novel biomarkers offer mechanistic insights into resistance pathways, they require specialized assays not routinely available in clinical practice. In contrast, tumor markers such as CEA and CA-19-9 represent readily accessible, cost-effective tools that can be measured serially throughout treatment to provide real-time assessment of therapeutic response.

The superior prognostic performance of CEA over CA-19-9 in our study—observed in both the continuous analysis (CEA-OS *p* = 0.002 vs. CA-19-9-OS *p* = 0.019) and the threshold-based analysis—contradicts with some previously reported studies. As previously proposed, CA-19-9′s shorter serum half-life compared to CEA may enable earlier detection of treatment response, and CA-19-9 functions as a selectin-binding adhesion molecule directly involved in tumor-vascular interactions and angiogenesis [15,27,28]. CA-19-9 is a cell surface glycoprotein involved in cellular adhesion, and cancer cells expressing this protein may present greater metastatic and invasive potential [29]. Moreover, the CA-19-9 may be involved in tumor cell-induced platelet aggregation, which promotes distant metastasis in CRC [30]. CA-19-9 has been shown to correlate with anti-VEGF therapy efficacy in mCRC [31], suggesting a mechanistic link between CA-19-9-mediated tumor-vascular interactions and angiogenic signaling. Given that regorafenib primarily targets VEGFR-mediated angiogenesis, CA-19-9 dynamics may more sensitively reflect regorafenib’s anti-angiogenic and anti-metastatic activity compared to CEA. However, the negligible correlation between CEA and CA-19-9 on-treatment dynamics in our cohort (Spearman rho = 0.056) suggests that these markers capture genuinely independent biological processes during regorafenib treatment, and the relative superiority of CEA may reflect its broader sensitivity to overall tumor burden changes rather than pathway-specific activity.

The prognostic significance of inflammatory parameters reflects systemic inflammation’s role in promoting tumor angiogenesis. Neutrophils and platelets may release pro-angiogenic factors and establish VEGF-independent angiogenic pathways [32]. Patients with elevated baseline inflammatory markers may harbor inherently aggressive tumor biology with redundant pro-angiogenic signaling that limits single-pathway VEGFR inhibition efficacy, consistent with prior inflammatory index studies in regorafenib-treated populations [14,33].

This study has several strengths. The staged analytical approach—beginning with continuous biomarker analysis and restricted cubic splines before proceeding to threshold-based stratification—addresses the methodological concern of information loss through dichotomization and provides a more complete characterization of the biomarker–survival relationship. The internal validation using bootstrap resampling and leave-one-out cross-validation provides transparency regarding cutoff stability, a step often omitted in similar biomarker studies. The evaluation of tumor marker dynamics at two timepoints provided insight into treatment response trajectories. Additionally, our real-world cohort reflects routine clinical practice, including patients with reduced starting doses, dose modifications, and varying performance status—populations often excluded from registration trials—enhancing external validity.

Several limitations warrant consideration. First, the retrospective, single-center design may limit generalizability and introduce selection bias. Second, the relatively small sample size (n = 61) limited statistical power, particularly for multivariable analyses, as evidenced by the loss of significance when adjusting for covariates despite adequate events-per-variable ratios. Third, bootstrap analysis revealed considerable instability in the optimized cutoff values, with wide 95% confidence intervals spanning much of the tested range for some marker–outcome combinations. While sensitivity analysis demonstrated that the AIC landscape contains broad plateaus of near-equivalent model fit around the selected thresholds, the specific cutoff values of +14.6% (CEA) and +23.5% (CA-19-9) should be considered approximate and require external validation before any clinical application. Fourth, imaging intervals varied in this retrospective setting, which may have introduced informative assessment bias for PFS. Fifth, the modest discriminatory ability observed in ROC analysis (AUC 0.55–0.65) suggests that tumor marker dynamics alone may not be sufficient for clinical decision-making. Finally, the observational nature of this study precludes definitive causal inferences.

## 5. Conclusions

These exploratory findings demonstrate that early on-treatment dynamics of both CEA and CA-19-9 show a continuous, linear association with survival in regorafenib-treated mCRC, with CEA percentage change showing the strongest association with overall survival (HR 1.007 per 10% increase, *p* = 0.002). Threshold-based stratification at +14.6% (CEA) and +23.5% (CA-19-9) provides clinically interpretable groupings that are significant for PFS in univariable analysis, though internal validation reveals the inherent instability of data-driven cutoff optimization in small samples. The negligible correlation between CEA and CA-19-9 dynamics (Spearman rho = 0.056) suggests these markers capture independent biological information during treatment.

These results provide preliminary evidence supporting further investigation of serial tumor marker monitoring during regorafenib therapy, though prospective validation in larger, independent cohorts—ideally including datasets such as the CORRECT trial—is essential before any clinical utility can be established. Future studies should evaluate whether continuous tumor marker dynamics provide incremental prognostic value beyond established clinical factors and whether treatment modifications guided by early marker trajectories can improve outcomes in this challenging patient population.

## Figures and Tables

**Table 1:** Patient Characteristics, Tumor Marker Dynamics, and Response Patterns (N = 61).

Characteristics	Value
**Demographics (*N* = 61)**	
Age, years—median (range)	62 (33–87)
Male gender, *n* (%)	37 (60.7%)
**Primary tumor sidedness, *n* (%)**	
Left-sided primary	39 (63.9%)
Rectum	12 (19.7%)
Right-sided primary	10 (16.4%)
**Mutational status, *n* (%)**	
RAS mutant	40 (65.5%)
BRAF mutant	1 (1.6%)
**Metastatic sites, *n* (%)**	
Liver	41 (72.1%)
Lung	19 (31.1%)
Peritoneum	6 (9.8%)
Other	3 (4.9%)
**Treatment History, *n* (%)**	
Third-line regorafenib	36 (59.0%)
≥Fourth-line regorafenib	25 (41.0%)
**Prior anti-EGFR therapy (RAS WT), *n* (%)**	15/21 (71.4%)
**CEA Levels (ng/mL)**	
Baseline—median (IQR)	56.5 (17.9–247)
After third cycle—median (IQR)	99.8 (24.8–335)
**CA-19-9 Levels (U/mL)**	
Baseline—median (IQR)	58.1 (24.4–272)
After third cycle—median (IQR)	113 (33.7–475)

CEA, carcinoembryonic antigen; CA-19-9, carbohydrate antigen 19-9; IQR, interquartile range; EGFR, epidermal growth factor receptor; VEGF, vascular endothelial growth factor; WT, wild-type.

**Table 2:** Univariate and multivariate Cox regression for factors affecting the progression-free and overall survival.

Factor	Univariate PFS HR (95% CI), *p* Value	Multivariate PFS HR (95% CI), *p* Value	Univariate OS HR (95% CI), *p* Value	Multivariate OS HR (95% CI), *p* Value
**Gender (Female vs. Male)**	1.14 (0.67–1.93) *p* = 0.632	-	1.15 (0.69–1.93) *p* = 0.593	-
**RAS/RAF mutational status (Wild-type vs. Mutant)**	0.83 (0.48–1.43) *p* = 0.510	-	0.70 (0.41–1.19) *p* = 0.191	-
**Primary tumor sidedness**				
Rectum	Ref	-	Ref	-
Right Colon	0.53 (0.22–1.28) *p* = 0.158	-	0.71 (0.30–1.67) *p* = 0.438	-
Left Colon	0.87 (0.44–1.73) *p* = 0.697	-	0.87 (0.45–1.68) *p* = 0.680	-
**Regorafenib treatment line (≥4th line vs. 3rd line)**	1.44 (0.85–2.45) *p* = 0.171	-	1.44 (0.85–2.44) *p* = 0.179	-
**CEA cutoff (above vs. below)**	2.06 (1.17–3.62) *p* = 0.013	1.71 (0.80–3.62) *p* = 0.164	1.67 (1.01–2.80) *p* = 0.050	1.81 (0.93–3.54) *p* = 0.079
**CA-19-9 cutoff (above vs. below)**	1.88 (1.05–3.36) *p* = 0.033	1.32 (0.61–2.87) *p* = 0.478	1.26 (0.75–2.12) *p* = 0.390	0.87 (0.44–1.71) *p* = 0.692

HR = Hazard Ratio; 95% CI = 95% Confidence Interval; PFS = Progression-Free Survival; OS = Overall Survival; CEA = Carcinoembryonic Antigen; CA-19-9 = Carbohydrate Antigen 19-9; Ref = Reference category; - = Not included in multivariable.

## Data Availability

The datasets generated during the study are available from the corresponding author upon reasonable request.

## Abbreviations

The following abbreviations are used in this manuscript:

AIC	Akaike Information Criterion
AST	Aspartate Aminotransferase
AUC	Area Under the Curve
CA-19-9	Carbohydrate Antigen 19-9
CEA	Carcinoembryonic Antigen
CI	Confidence Interval
CIPI	Cancer-Inflammation Prognostic Index
cmDNA	Circulating methylated DNA
CRC	Colorectal Cancer
CRP	C-Reactive Protein
ECOG	Eastern Cooperative Oncology Group
EGFR	Epidermal Growth Factor Receptor
HR	Hazard Ratio
IQR	Interquartile Range
mCRC	Metastatic Colorectal Cancer
MSI-H	Microsatellite Instability-High
MSS	Microsatellite-Stable
OS	Overall Survival
PDGF-AA	Platelet-Derived Growth Factor AA
PFS	Progression-Free Survival
RECIST	Response Evaluation Criteria in Solid Tumors
Ref	Reference category
ROC	Receiver Operating Characteristic
SD	Standard Deviation
VCAM-1	Vascular Cell Adhesion Molecule 1
VEGF	Vascular Endothelial Growth Factor
WT	Wild-Type
